# PIAS3 modulate HIV-1 integrase SUMOylation

**DOI:** 10.1186/1742-4690-8-S2-P4

**Published:** 2011-10-03

**Authors:** Guillaume Beauclair, Joris Paris, Fabien Magne, Alessia Zamborlini, Ali Saϊb

**Affiliations:** 1 CNRS UMR7212, INSERM U944, Institut Universítaire d'Hématologie-Uníversíté Paris7 Diderot, 75475 Paris, France; 2 Conservatoire des Arts et Métiers, Paris, France

## Background

HIV-1 hijacks cellular machineries to replicate. Post-translational modifications, like acetylation, phosphorylation or ubiquitination are not exception. We are interested in studying the interplay between HIV-1 and the SUMOylation pathway.

SUMOylation consists in the covalent attachment of SUMO (Small Ubiquitin-like Modifier) proteins to a lysine residue of a target protein, often found within a consensus motif (ΨKxD/E, where Ψ is a hydrophobic residue, x is any amino acid, D or E is an acidic residue). SUMO proteins share structural similarities with ubiquitin, and are conjugated to the substrate by an analogous but distinct enzymatic cascade requiring the sequential action of E1 activating enzyme (SAE1/SAE2 heterodimer), E2 conjugating enzyme (Ubc9), and E3 ligases. So far, several E3 SUMO ligases have been characterized, such as nuclear pore complex component Ran-binding protein 2 (RanBP2), protein-inhibitor of activated STAT (PIAS) family proteins, etc. Despite that Ubc9 can directly transfer SUMO moieties to acceptor lysine residues, E3 SUMO ligases promote specificity and/or efficiency of SUMO conjugation. We have recently shown that HIV-1 integrase, the viral enzyme that is responsible for viral genome integration into host cellular chromosome, is SUMOylated. Virions harboring a SUMOylation-defective integrase are less infectious than WT HIV-1, indicating that this modification is important for efficient viral replication [1].

## Materials and methods and results

To better characterize the involvement of SUMOylation during HIV-1 replication, we addressed the role of E3 SUMO ligases in the conjugation of SUMO to integrase.

By immunoprecipitation and GST pull-down approaches, we establish that HIV-1 integrase interacts with a PIAS family member protein, PIAS3. By immunofluorescence and confocal microscopy, we show that integrase and PIAS3 colocalise. Moreover, by Ni^2+^ pull-down in denaturing conditions, we demonstrate that PIAS3 increases integrase SUMOylation. We also analyzed the effect of PIAS3 over-expression in virus-producing or target cells and did not find a significant variation of viral infectivity.

## Conclusions

We find that one of the proteins of the PIAS family, PIAS3, interacts with HIV-1 integrase and increases its SUMOylation.
